# Tagging incidental finding of fatty liver on ultrasound: A novel intervention to improve early detection of liver fibrosis

**Published:** 2021-09-29

**Authors:** Navroop Nagra, Rubal Penna, Danielle La Selva, David Coy, Asma Siddique, Blaire Burman

**Affiliations:** ^1^Digestive Disease Institute, Virginia Mason Medical Center, Seattle, WA 98101, United States; ^2^Department of Radiology, Virginia Mason Medical Center, Seattle, WA 98101, United States

**Keywords:** nonalcoholic fatty liver disease, nonalcoholic steatohepatitis, risk factors, ultrasound, liver fibrosis

## Abstract

**Background:**

It is not uncommon to see that a large proportion of patients with cirrhosis due to nonalcoholic steatohepatitis never had any prior evaluation or diagnosis of liver disease, and most of the times their first clinical presentation is decompensated cirrhosis. Acknowledging incidental finding of fatty liver on abdominal imaging and identifying patients at risk of having advanced liver fibrosis may help in preventing its progression to cirrhosis.

**Aim:**

We aimed to increase acknowledgement and improve evaluation of steatosis through radiology recommendation to consider hepatology referral, and to identify the predictors of hepatology referral and significant fibrosis.

**Methods:**

We performed a retrospective study of 812 patients with hepatic steatosis tagged on ultrasound (US), over 18 months, at a single center. Patients with secondary causes of fatty liver were excluded from the study. We evaluated the yield of this intervention and factors correlated with hepatology referral and presence of significant fibrosis.

**Results:**

Diagnosis of fatty liver was acknowledged for 69% of patients with tagged US, although only 29% were ultimately seen by hepatology. Patients who had US ordered by a primary care provider (PCP) were more likely to have hepatology evaluation (64.8% vs. 56.9%, *P* = 0.0183). Sixty-six percent of patients seen by hepatology had elevated alanine transaminase (ALT) compared to 52% not seen by hepatology (*P* < 0.0005). Among patients further evaluated, 53% underwent staging, and 18% had ≥stage 2 (F2) fibrosis. Type II diabetes correlated with significant to advanced fibrosis (43.5% vs. 21.4%, *P* = 0.0357), while ALT and Body Mass Index did not.

**Conclusions:**

Tagging US reports led to clinical acknowledgement of fatty liver in 7 of 10 patients, although fewer than 1 in 3 had further hepatology evaluation. Of those who underwent staging for incidentally noted steatosis, 18% had significant fibrosis, suggesting that we are failing to evaluate patients with potentially advanced liver disease.

**Relevance for Patients:**

Identifying incidental finding of fatty liver on US provides a unique opportunity in diagnosing liver fibrosis at an early stage and can help prevent its progression to cirrhosis. PCP should consider using noninvasive scoring systems on a regular basis to assess the risk of fibrosis in patients with fatty liver, and timely referral to hepatology should be provided in patients at high risk of having advanced fibrosis.

## 1. Introduction

Nonalcoholic fatty liver disease (NAFLD) has become a global epidemic and is the most common liver disease in the United States [1]. The rising rate of fatty liver coincides with worldwide epidemics of obesity, type II diabetes (DMII), and metabolic syndrome. NAFLD is characterized by fatty changes in the liver in the absence of significant alcohol intake (<20 g/day for women and <30 g/day for men) and is sub-classified into nonalcoholic fatty liver (NAFL) and nonalcoholic steatohepatitis (NASH) [2]. With regard to liver outcomes, NAFL, or simple steatosis, is considered a benign condition with low likelihood of progressive liver disease or damage. However, NASH, the clinically significant form of NAFLD, is defined by steatohepatitis or inflammation which can lead to progressive liver fibrosis, cirrhosis, and eventually liver-related disease and death [3,4]. Once thought “benign,” NASH has become a leading etiology of cirrhosis, hepatocellular carcinoma (HCC), and indication for liver transplant. HCC is one of the fastest rising cancers worldwide [5]. All forms of fatty liver are associated with metabolic complications, namely, insulin resistance, and both increased risk of cardiovascular disease and death [6].

To date, there are no screening guidelines for fatty liver [7]. Most often NAFLD is diagnosed incidentally when patients are noted to have elevated liver enzymes or an echogenic liver on abdominal ultrasound (US). When steatosis is identified on US, various scoring systems such as NAFLD fibrosis score (NFS) and fibrosis 4 (FIB-4) index can be used to assess patients at risk of having advanced fibrosis, and to decide which patients need further workup; however, these scoring systems have low positive predictive value. NASH by definition is a histopathological diagnosis. Liver biopsy is required to truly differentiate NASH from benign steatosis but the invasive nature of this procedure precludes its routine use [8,9]. Magnetic Resonance Elastography (MRE), Fibroscan, and US shear wave elastography are helpful in assessing degree of liver fibrosis and can differentiate benign fatty liver from NASH with high accuracy once other secondary causes of hepatic steatosis are ruled out. Stage of fibrosis is the most clinically relevant data point and strongest determinate of liver-related morbidity and mortality [10].

A high proportion of patients presents with advanced liver disease secondary to NASH that was either not diagnosed or not evaluated previously. Further, we know that hepatic steatosis is frequently identified on imaging performed for reasons other than suspicion of liver disease, that is, incidentally. For these patients, it is unknown if follow-up occurs or what proportion ends up having clinically significant liver disease. Given that NASH and even fibrosis is reversible with appropriate intervention if caught before development of cirrhosis, early recognition is essential for preventive care [11].

We know that for various medical conditions, failure to follow-up on specific imaging recommendations can result in delayed and sub-optimal care. Prior studies have suggested that over 10% of patients with incidental steatosis noted on imaging (done for non-hepatic reasons) ended up having advanced fibrosis on further evaluation [12]. It was also shown that documentation of “hepatic steatosis” within the impression section of the radiology report was associated with a higher likelihood of acknowledgment by primary providers [12]. In this study, we conducted a novel intervention to “tag” every US where liver steatosis was identified with a recommendation “consider hepatology referral” within the impression section of the radiology report. We hypothesized that “tagging” steatosis in this manner would improve yield of diagnosis and subsequent evaluation. Here we present the yield of this intervention: what proportion of patients were eventually seen in hepatology clinic, and of those seen, what proportion had stage F2 or higher fibrosis based on invasive or non-invasive staging and factors correlated with advanced liver disease.

## 2. Materials and Methods

The radiology and hepatology departments at Virginia Mason Medical Center collaborated on a pilot project to “tag” every US report by bringing attention to hepatic steatosis in the impression section of the radiology report as follows: “Echogenic liver, most likely reflecting steatosis. Liver steatosis (fatty liver) can be an incidental benign finding, but in some individuals, it can be caused by non-alcoholic steatohepatitis (NASH) or other causes of chronic liver dysfunction. If the patient has not already been evaluated, consider hepatology consultation.” We conducted a retrospective chart review of all patients who underwent abdominal US for any reason between April 2016 and September 2017 and had this tag.

**Figure 1:**
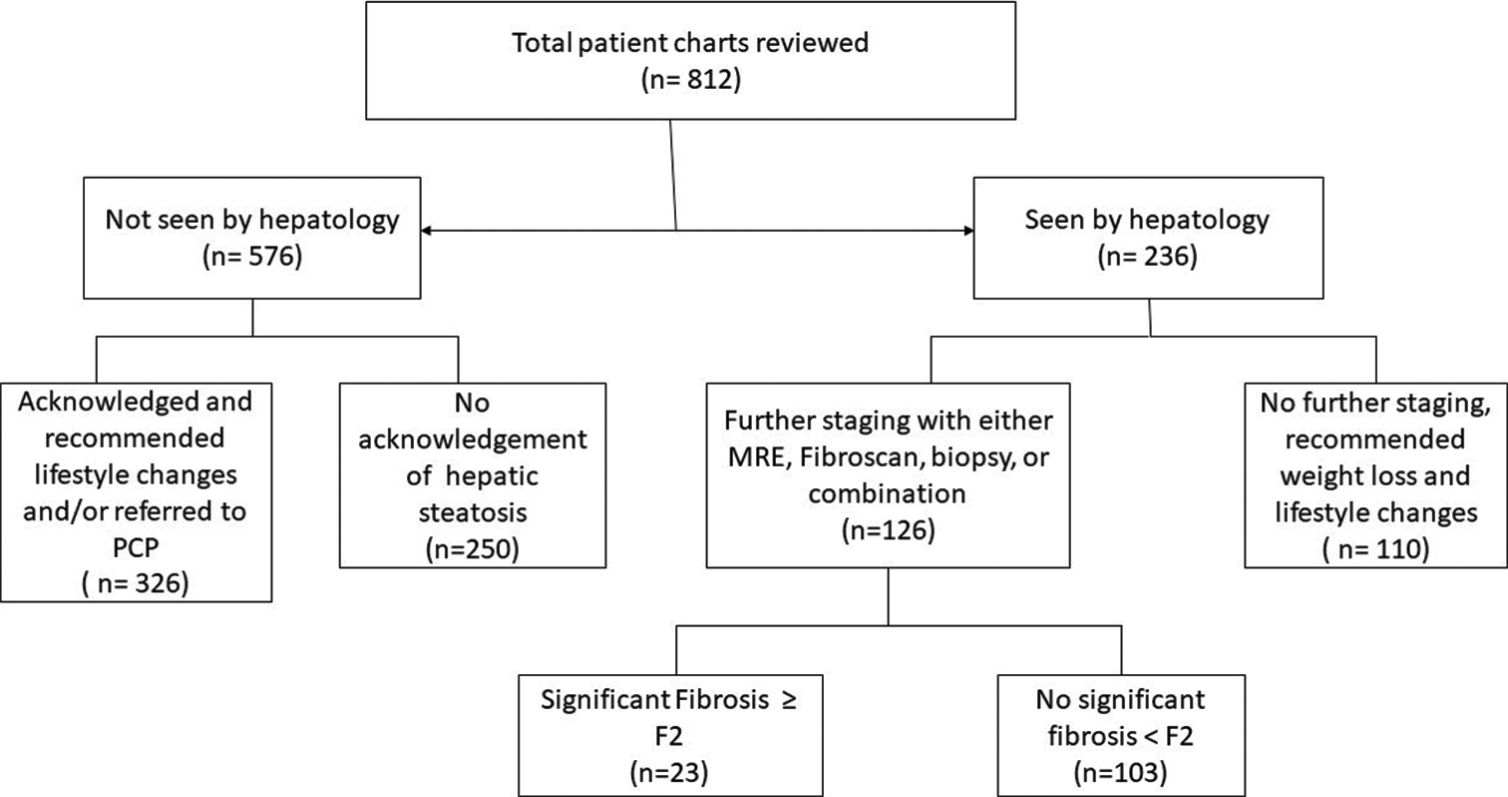
Incidentally identified hepatic steatosis: diagnosis, referral, and evaluation

A total of 812 patients with incidental hepatic steatosis were identified, and their electronic medical records were reviewed up to August 2019. Hence, time of review from date of report was at least 24 months to determine if fatty liver was acknowledged, if the recommendation for hepatology referral was made, and if patients were ultimately seen in hepatology clinic (Figure 1). Baseline demographics and metabolic risk factors were gathered. Predominant ethnicity in the cohort was white. The presence of DMII was defined as Hba1c of ≥ 6.5% or random blood glucose on serum chemistry or finger stick to be ≥ 200 mg/dL. The indication for location of US (i.e., emergency department [ED], hospital, and clinic) and ordering provider was documented. For those seen by hepatology, we assessed what subsequent evaluation they had including staging fibrosis with either transient elastography (FibroScan), MRE, and/or liver biopsy. Of those who had staging, we evaluated what proportion had stage F2 or higher fibrosis (significant fibrosis) and identified predictors of advanced fibrosis. For this study, normal alanine transaminase (ALT) was defined as <35 U/L for men and <25 U/L for women, which are the parameters used in our medical center and consistent with American College of Gastroenterology guidelines [13].

We excluded patients with significant alcohol use based on chart review and social history. Significant alcohol use was defined as >20 g/day for women and >30 g/day for men. Patients with use of medications known to induce steatosis (i.e., methotrexate, tamoxifen, and amiodarone) were excluded from the study. Patients with other chronic liver disease such as viral hepatitis B or C, hemochromatosis, primary sclerosing cholangitis, primary biliary cholangitis, Wilson’s disease, and/or Alpha 1 antitrypsin deficiency were also excluded from the study.

Characteristics of patients with incidental liver steatosis who were evaluated in hepatology clinic were compared to those who were not referred. Second, characteristics of patients who had staging and were found to have clinically significant (≥F2) to advanced (F3, F4) fibrosis were compared to those without significant liver fibrosis (F0, F1).

### 2.1. Statistical analysis

Continuous variables were analyzed using the Student’s *t*-test and categorical variables using the Chi-square test or Fisher’s exact test. For each calculation, *P* < 0.05 was used as the threshold for statistical significance.

The study was approved by Virginia Mason Institutional Review Board (IRB19-085).

## 3. Results

After the application of exclusion criteria, 812 patients with incidental findings of hepatic steatosis on US were identified. Median age of patients was 54 (interquartile range 42 – 61) years, and 432 (53.2%) were women. In terms of body mass, 9.7% had body mass index (BMI) <24.9, 28.9% had BMI 25 – 29.9, 31.8% had a BMI 30 – 34.9, 13.9% had BMI 35 – 39.9, and 15.4% had BMI of >40. Based on above criteria, 23.2% had DMII. Majority of patients had private insurance (73.4%) as compared to Medicare or Medicaid (22%). In terms of indication, 28.2% had US to evaluate “elevated liver enzymes” while 13.7% had US to evaluate abdominal pain, and majority (58.1%) were for other reasons with no suspicion of hepatobiliary disease. Primary care providers (PCP) ordered most (59.5%) of the scans, while 6.6% were ordered by gastroenterologists (GI), 23.6% by a medical subspecialist other than GI, 5.8% by surgery, and 4.9% through the ED or urgent care. Overall 43.3% had normal ALT and 56.7% had elevated ALT (Table 1).

**Table 1:** Comparison between patients with fatty liver, with and without hepatology referral

	Hepatology	Not seen by hepatology	*P*-value
Demographics	*n*=236	*n*=576	
Age, m±SD	53.1±14.3	55.4±13.8	0.0405
Sex - Female, *n* (%)	129 (54.7)	303 (52.6)	-
Sex - Male, *n* (%)	107 (45.3)	273 (47.4)	0.6422
BMI	*n*=234	*n*=563	
<24.9 - *n* (%)	21 (9.0)	57 (10.1)	0.6953
25 – 29.9 - *n* (%)	70 (29.9)	161 (28.6)	0.7319
30 – 34.9 - *n* (%)	73 (31.2)	181 (32.1)	0.803
35 – 35.9 - *n* (%)	32 (13.7)	79 (14.0)	0.8946
>40 - *n* (%)	38 (16.2)	85 (15.1)	0.6685
Type II Diabetes status	*n*=234	*n*=531	
DMII present, *n* (%)	46 (19.7)	132 (24.9)	0.1372
Insurance	*n*=234	*n*=564	
Self pay, *n* (%)	1 (0.4)	35 (6.2)	< 0.0001
Medicare, *n* (%)	33 (14.1)	143 (25.4)	0.0005
Private, *n* (%)	200 (85.5)	386 (68.4)	< 0.0001
Indication	*n*=236	*n*=576	
Liver, *n* (%)	111 (47.0)	117 (20.3)	< 0.0001
Biliary, *n* (%)	26 (11.0)	86 (14.9)	0.1471
Other *n* (%)	99 (41.9)	373 (64.8)	< 0.0001
ALT	*n*=234	*n*=530	
Normal*, *n* (%)	79 (33.8)	252 (47.5)	-
High, *n* (%)	155 (66.2)	278 (52.5)	0.0005
Platelets	*n*=234	*n*=529	
Normal**, *n* (%)	223 (95.3)	503 (95.1)	-
Low, *n* (%)	11 (4.7)	26 (4.9)	0.8990
Ordering MD	*n*=232	*n*=576	
Primary care, *n* (%)	153 (64.8)	328 (56.9)	0.0183
GI, *n* (%)	25 (10.2)	29 (5.0)	0.0047
Surgery, *n* (%)	12 (5.1)	35 (6.1)	0.7403
Medical subspecialty other than GI, *n* (%)	46 (19.5)	145 (25.2)	0.1197
ED or urgent care, *n* (%)	1 (0.4)	39 (6.8)	<0.0001

* Normal ALT defined as <25 U/L for women as <35 U/L for men

** Normal platelets defined as ≥150×10^9^/L

**Figure 2:**
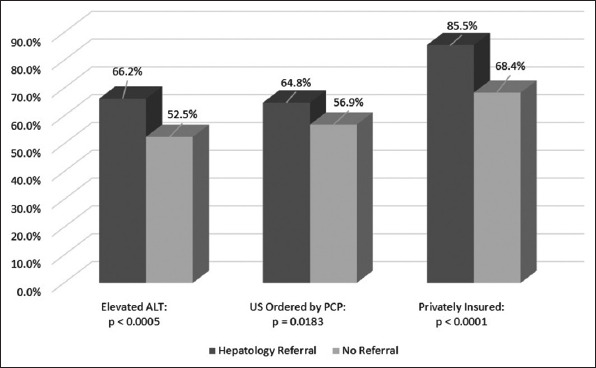
Factors correlated with hepatology referral

Findings of hepatic steatosis were acknowledged in 512 patients (69.2%) by the ordering provider or PCP but not everyone was referred to or seen by hepatology. Two hundred and thirty-six (29%) of all patients with steatosis were seen by hepatology (Tables 1 and 2). Patients with elevated ALT were more likely to be referred to hepatology than those with normal liver enzymes (66.2 vs. 52.5%, *P* < 0.0005; Figure 2). Majority of patients seen by hepatology were privately insured (85% vs. 68.4%, *P* < 0.0001; Figure 2). Patients who were self-insured were much less likely to be referred and/or seen by hepatology (0.4 – 6.2%, *P* < 0.0001). Patients with higher BMIs were not more likely to be referred. Patients with DMII were also not more likely to be referred, and in fact a lower percent of those with diabetes (19.7 vs. 24.9%. *P* = 0.137) were seen in hepatology clinic. Nearly half (47%) of patients seen by hepatology had an US ordered to evaluate elevated liver enzymes or suspicion for liver disease, as compared to 20.3% patients not seen by hepatology (*P* < 0.0001). Further, patients whose US was ordered by primary care or GI were more likely to be seen by hepatology (64.8% vs. 56.9%, *P* = 0.0183 and 10.2% vs. 5.0%, *P* = 0.004, respectively; Table 1).

**Table 2:** Comparison between patients with fatty liver with no/minimal versus significant fibrosis

	Staging≥F2	Staging<F2	*P*-value
Demographics	*n*=23	*n*=103	
Age, m±SD	56.3±10.7	53.1±14.4	0.3071
Sex - Female, *n* (%)	9 (39.1)	49 (47.6)	-
Sex - Male, *n* (%)	14 (60.9)	54 (52.4)	0.4969
BMI			
<24.9 - *n* (%)	1 (4.3)	11 (10.7)	0.6934
25 – 29.9 - *n* (%)	5 (21.7)	28 (27.2)	0.7938
30 – 34.9 - *n* (%)	10 (43.5)	37 (35.9)	0.6340
35 – 39.9 - *n* (%)	2 (8.7)	15 (14.6)	0.7362
>40 - *n* (%)	5 (21.7)	12 (11.7)	0.1955
Type II diabetes (DMII) status			
DMII present, *n* (%)	10 (43.5)	22 (21.4)	0.0357
Indication			
Liver, *n* (%)	12 (52.2)	51 (49.5)	0.8176
Biliary, *n* (%)	5 (21.7)	8 (7.8)	0.0609
Other/Incidental, *n* (%)	6 (26.1)	44 (42.7)	0.1634
ALT			
Normal,* *n* (%)	6 (26.1)	35 (34.0)	-
High, *n* (%)	17 (73.9)	68 (66.0)	0.6236
Platelets			
Normal,** *n* (%)	19 (82.6)	101 (98.1)	-
Low, *n* (%)	4 (17.4)	2 (1.9)	0.0102
Lipid profile	*n*=23	*n*=81	
Elevated LDL,*** *n* (%)	12 (52.2)	48 (59.3)	0.6345
Elevated triglycerides,*** *n* (%)	17 (73.9)	59 (72.8)	0.9184

* Normal ALT defined as <25 IU/L for women and <35 IU/L for men

** Normal platelets defined as ≥150×10^9^/L

*** Elevated LDL defined as ≥130 mg/dL

****Elevated triglycerides defined as ≥150 mg/dL

**Figure 3:**
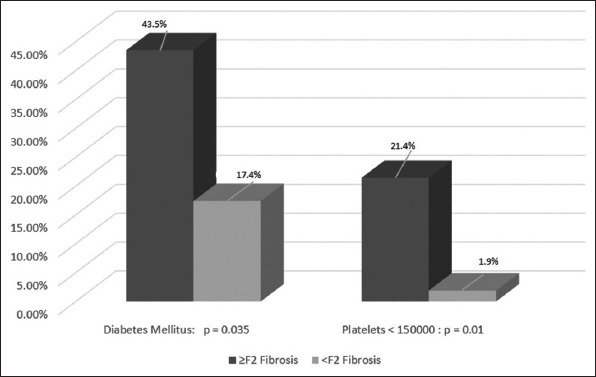
Factors correlated with significant fibrosis

Out of 236 patients who were further evaluated in hepatology clinic, 126 patients (53%) underwent staging in the form of transient elastography (FibroScan), MRE, and/or liver biopsy (Table 2). Twenty-three out of 126 patients (18.2%) were found to have Stage F2 or higher stage fibrosis, which we considered clinically significant fibrosis based on increased risk of liver-related morbidity and mortality noted in natural history studies [10]. Of those with staging, the presence of DMII was strongly correlated with clinically significant fibrosis in this study (43.5% vs. 21.4%, *P* = 0.0357; Figure 3). Low platelets were strongly correlated with ≥ Stage F2 (17.4% vs. 1.9% *P* = 0.0102; Figure 3). Elevated ALT level was not a significant predictor of significant fibrosis. Elevated low-density lipoprotein cholesterol and triglycerides were also not correlated with clinically significant fibrosis.

## 4. Discussion

Our study demonstrates that fatty liver is a common incidental finding and that even with explicit radiology recommendation to pursue hepatology referral, the majority of those found to have hepatic steatosis did not get further evaluation. Of those who did, 20% were found to have significant (F2) to advanced fibrosis (F3, F4) and hence at risk for morbidity and mortality secondary to liver disease. Despite prompting in the impression section of radiology reports, many providers, and likely patients, do not take heed the suggestion. There is room for improved education regarding potential severity of fatty liver and benefit of further assessment and management.

Most patients with NAFLD are followed by primary care, and identifying those with significant fibrosis, who might benefit from specialist referral, is challenging. Based on the natural history of fatty liver disease, up to 75 – 80% of these cases are likely benign [14]. Furthermore, there are multifaceted limitations to accessing subspecialty care in United States, and advanced imaging techniques are expensive and not widely available. Pathways that help PCP “triage” the overwhelming numbers of individuals with fatty liver to those who are at low risk and can be monitored, along with lifestyle modifications, versus those who should be referred for further testing are emerging.

Noninvasive scoring systems are one such method available to primary providers to identify which patients are more likely to have advanced liver disease. These include the NFS as FIB-4 index. Both have a high negative predictive value for advanced fibrosis, though many patients fall into the “indeterminate zone,” and these models have not been extensively validated [15]. Liver biopsy remains the gold standard for diagnosis of NASH and is essential in cases of diagnostic uncertainty. That said, our ability to detect patients with Stage 2 or greater fibrosis is most clinically relevant, given the majority of simple steatosis cases do not progress, and hepatic fibrosis is the strongest predictor of risk for liver-related adverse outcomes. Noninvasive radiographic evaluation of fibrosis involves measurement of elastic shear wave propagation through liver tissue. The best validated imaging methodologies include US shear wave elastography and transient elastography (e.g., FibroScan) with a sensitivity of 85% for detecting advanced fibrosis, while MRE has a similar 86% sensitivity [16,17].

The previous studies have indicated that hepatic steatosis is a common incidental finding on imaging and, in this setting, often not acknowledged as a diagnosis that requires further evaluation. A study by Kutaiba *et al*. showed that of patients with suspected renal colic who underwent imaging in the ED setting, 26% of the 1290 patients had hepatic steatosis on CT, and only 28% of those patients had fatty liver ultimately documented in the radiology report [18]. This omission highlights multiple gaps in the reporting and evaluation of hepatic steatosis among radiologists and emergency providers alike. A follow-up prospective survey explored preferences and perspectives of ED physicians regarding reporting of incidental hepatic steatosis. Fewer than half of ED physicians reported they would discuss fatty liver with patients, and 30% felt that radiology reporting of steatosis was irrelevant in the emergency setting. Majority reported that an incidental finding such as fatty liver would have more significance if mentioned in the conclusion section [19]. In a study published in 2016 by Wells *et al.*, 10.7% of patients who had CT in the ED were incidentally found to have hepatic steatosis, but 74% of family physicians were unaware of the findings, and 13% who were aware did not pursue any further workup [20].

A single center study by Wright *et al*. demonstrated that newly identified steatosis on CT was documented in the medical record in only 23% of patients in a 14-month follow-up period. Further, no patients were referred for specialist evaluation or liver biopsy. This was concerning given when a NFS was calculated, 11% had high risk for advanced fibrosis but were lost to follow-up [12]. Placement of findings of steatosis in the “impression” section as compared to the body of the report significantly increased the likelihood of PCPs documenting fatty liver in the patients’ medical record (30.1 vs. 9.1%, *P* < 0.05). Accordingly, we hypothesized that “tagging” steatosis in the US report and specifically the impression section would improve the yield of diagnosis. Indeed, 69.2% had fatty liver acknowledged, most often by primary care, and this is higher than previous reports in the literature. However, fewer than 1 in 3 patients were referred to hepatology, as recommended, for further evaluation. Most exams were ordered by primary care or medical subspecialists, and relatively few were identified through the ED or acute care.

Risk factors for fatty liver and metabolic disease were common. In this study, 90% of patients with hepatic steatosis were overweight or obese, and 30% had a BMI ≥35; however, elevated BMI was not correlated with hepatology referral. While those with a BMI >40 who underwent staging were more likely to have significant fibrosis, this finding was not statistically significant. Nearly 1 in 4 individuals with steatosis on US had DMII. While, we know insulin resistance and presence of DMII is associated with a higher risk of NASH (vs. benign steatosis) and liver fibrosis [21]. Patients with DMII were not more likely to be referred for further evaluation in this study and in fact, a lower proportion were seen by hepatology (19.7% vs. 24.9%). For those who went on to have staging, 43.5% of those with significant to advanced fibrosis had DMII compared to 21.4% of those with the lower stage disease. Improving upstream identification, evaluation, and referral of those found to have steatosis with underlying DMII would greatly enhance the ability to detect advanced NASH cases at risk for cirrhosis and liver cancer.

It is not clearly known what proportion of patients with fatty liver have elevated transaminases. Studies have shown that up to 30% of patients with biopsy-proven NASH have normal ALT levels [22]. In this study, diagnosis of fatty liver was made by imaging and exclusion of regular alcohol use or other chronic liver diseases, not by elevated ALT. Of this cohort, ALT was elevated for the majority, but 40% had entirely normal liver enzymes. While we know the presence or degree of ALT elevation does not correlate with the diagnosis of NASH or severity of fibrosis [23], ALT elevation was strongly correlated with hepatology referral, and those with normal ALT were unlikely to be seen by hepatology. For those who underwent staging, ALT was not a predictor of fibrosis. Providers are likely to miss a significant number of individuals with severe NASH by limiting clinical evaluation to those with fatty liver and elevated transaminases.

There are several limitations to our study. First, while included patients had no prior diagnosis of fatty liver or evaluation of liver disease, given the indication for US was in some cases “elevated liver enzymes,” fatty liver may have been an expected finding. The majority of our patient population was white, so these results may not be generalizable. Despite our best efforts to exclude patients with other causes of fatty liver, such as alcohol and other chronic liver diseases, few patients may still have fatty liver due to secondary causes. It is also possible that providers discussed fatty liver with the patient, but this was never documented, or they made recommendations for further care that was not completed by the patient. Given the single-center nature of our study, certain institutional practices may be unique to our system. Finally, long-term follow-up would be needed to identify whether incidental NAFLD diagnosis and/or hepatology evaluation and staging improved clinical outcomes.

Patients with advanced NASH often present late with complications of cirrhosis and/or liver cancer. Lack of effective management contributes to poor outcomes. In our study, a novel radiology intervention to bring greater attention to the finding of hepatic steatosis did in fact improve provider acknowledgement compared to what is documented in the literature, though only one-third of patients were referred to hepatology, as recommended. Predictors of advanced NASH including DMII and higher BMI were not associated with hepatology referral, whereas elevated ALT was strongly correlated with referral. Based on these findings, incidental identification of fatty liver on US is a critical opportunity for diagnosis and downstream disease management; however, there is room to improve our pathways for evaluation and specialty care.
